# The acceptability of a therapist-assisted internet-delivered cognitive behaviour therapy program for the treatment of anxiety disorders in adolescents: a qualitative study

**DOI:** 10.1007/s00787-021-01903-6

**Published:** 2021-11-08

**Authors:** Katy Smart, Lydia Smith, Kate Harvey, Polly Waite

**Affiliations:** 1 School of Psychology, University of Southampton, Southampton, UK; 2 Faculty of Health and Life Sciences, Coventry University and University of Warwick, Coventry, UK; 3 School of Psychology and Clinical Language Sciences, University of Reading, Reading, UK; 4 Departments of Experimental Psychology and Psychiatry, University of Oxford, Oxford, UK

**Keywords:** Cognitive behavioural therapy, Online, Internet, Adolescent, Anxiety disorders, Qualitative

## Abstract

Computerized treatments have been shown to be effective in young people with anxiety disorders. However, there has been limited investigation into the experiences of adolescents in undertaking this treatment. This qualitative study explored adolescents’ experiences of being treated for an anxiety disorder, using an online intervention with therapist support, as part of a randomised controlled trial. Individual semi-structured interviews were conducted with thirteen adolescents aged between 13 and 17 years, who had received the online intervention. Data were analysed using reflexive thematic analysis. Two cross-cutting themes were identified: (i) Usability of the program, and (ii) Putting techniques into practice. Although the convenience of online sessions was recognised as positive, many of the adolescents expressed a preference for face-to-face sessions. The length of sessions and the large amount of reading involved was highlighted by most participants. The transdiagnostic nature of the content meant some elements of the programme were seen as less relevant. While many participants appreciated the ‘fun’ aspect of the program, others felt that certain aspects of the program were not suitable for their age group. Exposure, psychoeducation and cognitive restructuring were generally received positively, whereas responses to the use of relaxation and rewards were much more mixed. Our findings highlight the potential utility for online treatments delivered with therapist support. However, they also highlight how issues with both the format and treatment components can impact the acceptability of a program. As such, further work is warranted to improve the acceptability of online treatments for adolescents with anxiety disorders.

**Supplementary Information:**

The online version contains supplementary material available at 10.1007/s00787-021-01903-6.

## Introduction

Anxiety disorders are among the most common mental health difficulties experienced by adolescents [1]. Prevalence rates of anxiety disorders in adolescents are around 8–11% [2, 3], and appear to have increased over recent years [2]. Around a third of adolescents referred for the treatment of an anxiety disorder also experience a comorbid mood disorder[4]. Adolescent anxiety disorders are associated with significant impairments in social functioning, such as social competence, negativity in social relationships, loneliness and victimisation, and academic functioning, such as difficulty concentrating, performance in examinations, and problems attending school [5]. Furthermore, they are associated with later negative life outcomes in adulthood, such as anxiety and depression, drug dependence and educational underachievement [6]. Consequently, it is clear that effective interventions are vital for long-term outcomes.

Cognitive behaviour therapy (CBT) has a substantial evidence base for the treatment of anxiety disorders in children and adolescents and is recommended by the National Institute for Health and Care Excellence (NICE) guidelines as the first-line treatment approach [7]. Key components of CBT for the treatment of anxiety disorders are typically exposure to feared situations or stimuli in a graded way (such as through an exposure hierarchy), accompanied by anxiety management strategies, including psychoeducation about anxiety, cognitive restructuring to identify and challenge negative automatic thoughts and skills training such as relaxation or problem-solving (e.g., [8], 9). The most recent Cochrane review of CBT for anxiety disorders in children and adolescents (aged 2–19 years) found that remission rates for the primary anxiety disorder were greater following CBT versus wait list controls (49.4% and 17.8%, respectively) [10].

Although anxiety disorders can be treated effectively, only a minority of young people access specialist mental health services [2, 11, 12]. A recent systematic review examined the reasons given by young people for not seeking or accessing professional help for mental health problems. They identified a complex array of factors, with barriers including perceived social stigma and feelings of embarrassment, lack of accessibility (e.g., time, transport, cost), and a lack of available help [13]. Research also indicates that adolescents prefer to rely on themselves instead of seeking professional support for their difficulties [14]. Online interventions may, therefore, be a means of reducing barriers and engaging young people in therapy, enabling those who are unable or unwilling to engage in a face-to-face intervention to receive treatment [15, 16]. This advantage has been particularly evident during the COVID-19 pandemic, allowing individuals to access therapy without the potential health risks of sessions being in-person [17]. Delivery through an online program may also reduce therapist time and enable services to shorten waiting lists [18]. Although positive findings are emerging for the use of online interventions [19], there remain challenges that need to be addressed. Whilst some users value the anonymity and privacy afforded, others perceive this to be impersonal and there can be difficulties maintaining compliance [20].

The current study is focused on an online CBT program for adolescents with anxiety disorders, *BRAVE for Teenagers-ONLINE* [21]. Positive outcomes were demonstrated in the initial randomised controlled trial undertaken in Australia, with no significant differences found between the therapist-supported online version and face-to-face treatment [22]. By the 12-month follow-up, differences remained non-significant; with 78% and 81%, respectively, no longer meeting criteria for their primary diagnosis. This program was then evaluated in a randomised controlled trial within a routine clinical service within the National Health Service in the UK [23]. Although the proportion of adolescents who were free of their primary anxiety disorder post-treatment (40.0%) was broadly consistent with Spence et al.’s [22] findings at the 12-week assessment time point (34.1%), there was no significant difference post-treatment between the immediate treatment and waitlist groups in remission of primary anxiety disorder (Odds Ratio (OR) = 2.19, 95% CI 0.72–6.70). This suggests that within routine clinical services at least, it is crucial to understand the acceptability of such treatments to inform further treatment development.

The importance of user engagement in the design and development of new interventions has been increasingly recognised [24]. In particular, qualitative methodology can be used to investigate patient perspectives of therapy as part of a co-design process or to examine the acceptability of existing therapeutic interventions to increase acceptability and effectiveness. In a qualitative meta-synthesis of eight studies of user (adults and young people) experiences of computerised therapy for anxiety and depression, Knowles et al. [25] identified two themes: first, that the programme needed to be sensitive to the user as an individual (e.g., providing personalised and relevant material appropriate to their specific needs) and second, that the format could both provide a sense of ownership and control but also burden, as it places greater demands on the user to motivate themselves to login and complete the activities, in the absence of support from a therapist. Both these themes were endorsed in the two studies in the meta-synthesis that involved adolescents, receiving an online self-help program for psychological distress (aged 15–18 years; [26]) and subclinical depression (aged 14–21 years; [27]).

However, as far as we are aware there has been no qualitative research investigating the acceptability of treatments for young people using an online intervention for the treatment of anxiety disorders. Qualitative approaches provide rich, participant-driving data and are useful in the context of randomised controlled trials as part of a mixed method approach to explore a range of issues related to acceptability [28]. This may include the perceived value, benefits, harms or unintended consequences of the intervention, barriers and facilitators to engagement, perceived ‘active ingredients’, and who is most likely to respond positively to the intervention [29, 30]. Accordingly, the aim of this qualitative study was to explore the acceptability of a therapist-supported online CBT program for the treatment of an anxiety disorder in adolescents, to understand what they liked and disliked, their perceptions of different aspects of the program, and how the program might be improved.

## Methods

The study adopted the epistemological and ontological position that reality is considered subjective, derived from participants’ perspectives, and can be described in terms of the meanings that participants attach to experiences. To ensure rigour, the COREQ checklist [31] was used for reporting (see Online Resource). Ethical approval for the study was granted by the National Research Ethics Committee London – Brent (reference: 12/LO/0119) and the University of Reading Research Ethics Committee.

### Participants

Participants of this study had taken part in a randomised controlled trial of a therapist-supported Internet treatment for adolescent anxiety disorders in a UK NHS Child and Adolescent Mental Health Service (CAMHS) (trial registration number: ISRCTN79652741).

To be eligible for the trial, adolescents had to meet DSM-IV diagnostic criteria [32] for either generalized anxiety disorder, separation anxiety disorder, social anxiety disorder, specific phobia, panic disorder with or without agoraphobia or agoraphobia without panic disorder, and this had to be identified as the primary problem. Adolescents could receive psychotropic medication if it had been on a stable dosage for 2 months. Adolescents were excluded if they had psychotic symptoms, substance dependence, conduct disorder, an autistic spectrum disorder, learning problems that would interfere with their understanding and participation in the trial (based on school, clinic or parent information), had self-harmed within the previous month, were not able to understand and speak English at an age-appropriate level or were currently receiving any other therapy or treatment for anxiety. Participants were also required to have a computer and Internet access at home.

To be eligible for the qualitative study, the adolescent needed to have participated in the trial but there was no requirement relating to the number of sessions they had completed. Thirteen adolescents took part in this study, all of whom had completed at least eight (of the ten) sessions of the program. Table 1 presents demographic and clinical background information for each participant.

**Table 1:** Participants’ demographic and clinical characteristics

Pseudonym	Age^a^	Gender	Ethnicity	Parent’s occupation	Pre-treatment primary disorder (CSR)	Pre-treatment secondary disorders (CSR)	Treatment condition	Number of sessions	CGI-I
Matthew	13	Male	White British	Higher professional	Generalized anxiety disorder (5)	None	Immediate	10	Very much improved
George	14	Male	White British	Higher professional	Generalized anxiety disorder (6)	None	Immediate	10	Very much improved
Chloe	13	Female	White British	Higher professional	Generalized anxiety disorder (5)	None	Wait-list	10	Very much improved
Isabelle	14	Female	Other mixed background	Higher professional	Generalized anxiety disorder (5)	None	Wait-list	10	Very much improved
Zoe	14	Female	White British	Other employed	Social anxiety disorder (6)	Generalized anxiety disorder (5)	Immediate	10	Minimally improved
Clare	14	Female	White British	Higher professional	Social anxiety disorder (5)	None	Wait-list	10	Very much improved
Rosie	13	Female	White British	Higher professional	Social anxiety disorder (7)	None	Wait-list	10	No change
Lana	17	Female	White British	Other employed	Social anxiety disorder (7)	None	Wait-list	10	Very much improved
Emma	13	Female	Other mixed background	Other employed	Panic disorder and agoraphobia (7)	None	Immediate	10	Much improved
Oliver	16	Male	White British	Other employed	Agoraphobia (7)	Social anxiety disorder (5)	Immediate	10	Minimally improved
Jayne	17	Female	White British	Higher professional	Specific phobia (5)	None	Wait-list	10	Very much improved
Becky	16	Female	White British	Higher professional	Specific phobia (5)	None	Wait-list	8	No change
Amelia	13	Female	White British	Other employed	Specific phobia (6)	None	Immediate	10	Minimally improved

^a^Participant age at recruitment into the trial. CSR = clinician Severity Rating (ranging from 0 (absent or none) to 8 (very severely disturbing/disabling); to meet diagnostic criteria, the adolescent was required to have a CSR of 4 or more). CGI-I = Clinical Global Impression – Improvement scale (CGI-I) [49] (on a scale from 1 (very much improved) to 7 (very much worse); in line with previous studies (e.g., [50]), the data was recoded as a categorical variable (scores of 1 and 2 were ‘improved’ and scores of 3 to 7 were ‘not improved’).

### Sampling

Participants were approached to take part in the qualitative study at the time of their 6-month follow-up assessment in the clinic (*n* = 9) or if the 6-month follow-up assessment time point had passed, via an email to their parent/carer (*n* = 33). Sixteen young people who were eligible were not invited, either because families had opted out of further contact or we no longer had their up-to-date contact details. The email emphasised that we were keen to hear a range of views, including from young people who had started but not completed the program and/or the follow-up assessment. Of those approached face-to-face at the time of the follow-up assessment, all agreed to participate. Of the 33 sent an email invitation, 4 (12%) consented to participate in the interview. Twenty-seven individuals (82%) did not respond to the email, 1 person (3%) declined to take part due to increased levels of anxiety and the other young person (3%) did not take part as they no longer lived in the UK. Because participants for this study were drawn from a sample recruited to a randomised controlled trial, we intended to create a pool of eligible participants and purposively sample young people who were diverse on key demographic and clinical characteristics. However, in practice, our ability to purposively sample was limited by the number of young people declining to participate and constraints relating to time and capacity. To determine a reasonable point at which to cease data collection, we kept under review the extent to which each interview resulted in a refinement of our codes, and judged that the information generated in the final two interviews (of 13) had not led to any new codes or themes/subthemes [34].

### Intervention

The online treatment consisted of *BRAVE for Teenagers-ONLINE* [21], an internet-based treatment for adolescent anxiety. It consists of ten weekly sessions, each of approximately 60 min duration, and two booster sessions (1 month and 3 months following treatment) to consolidate skills. Sessions incorporate standard CBT anxiety management strategies including psychoeducation, relaxation training, recognition of the physiological symptoms of anxiety, cognitive strategies of coping self-talk and cognitive restructuring, graded exposure, use of rewards and problem solving. Each participant was supported by a therapist who encouraged them to complete sessions on time, emailed individualised feedback after each session, and provided a telephone call mid-way through to help them plan their exposure hierarchy (the ‘BRAVE ladder’).

### Procedure

Participants were all referred by primary and secondary care services to the Berkshire NHS Foundation Trust CAMHS Anxiety and Depression Pathway. As part of the routine CAMHS assessment, they were systematically assessed to establish whether they had an anxiety disorder and/or any other diagnoses and to identify the primary problem. This was determined through the Anxiety Disorders Interview Schedule (child and parent-report) (ADIS-C/P) [33]. This is a structured interview, with good psychometric properties [35], designed to assess for current DSM-IV anxiety disorders and common comorbid disorders. If the adolescent met eligibility criteria for the trial, both parents and adolescents were given information sheets about the study. The young person and their parent then met with a member of the clinical team for a further appointment to discuss the results of the assessment and the RCT. If they chose to take part in the study, both provided informed consent. If they chose not to take part, they were placed on the waiting list for treatment within CAMHS. The recruitment process for the trial is described in more detail in the paper reporting on the trial’s outcomes [23]. Recruitment to the study was from July 2012 to December 2013 and all follow-up assessments were completed by December 2014. Qualitative interviews were conducted between November 2014 and March 2015.

Interviews took place after the final 6-month follow-up assessment so that they did not inadvertently act as part of the intervention. The mean length of time before a participant took part in this study was 11 month post-treatment. Data were collected using individual semi-structured, face-to-face interviews with participants. Before the interview took place, participants were told the research aims of the study and that all data would be audio recorded and anonymised. Informed written consent was obtained from all adolescents aged 16 years or older. If adolescents were 15 years or younger, they provided written assent and their parent provided written consent. Nine participants were interviewed in a private room in the clinic (for four participants, this was immediately after their 6-months follow-up assessment) and four were interviewed in their home. The interviews lasted between 33.2 and 60.8 min (mean = 44.3 min) and were audio recorded before later being transcribed verbatim. As visual narrative methods can facilitate recall, be a helpful tool to gain a deeper level of insight than asking questions alone [36] and have been successfully used in adolescent mental health research [37], screenshots of webpages from the intervention were shown to the participants during the interview. The interviewers made field notes during and after the interviews. During the interviews, this included thoughts related to the participants’ responses and ideas for additional questions. Following the interviews, notes were used to capture details related to the interview, and reflections around the interview, including the interviewer’s emotional reactions and initial ideas for data analysis. The interviews were conducted either by KS or LS (both female), who had both completed an undergraduate degree and were, at the time, MSc students at the University of Reading. They undertook training in qualitative methods as part of their MSc and completed this research because of an interest in in clinical psychology and in partial fulfilment of their MSc qualification. They had not been involved in the randomised controlled trial evaluating the program and had no pre-existing relationship with the participants involved in the research or the program itself. Other members of the research team were KH, a qualitative researcher who had no involvement in the RCT, and PW, a clinical psychologist and researcher who had led the trial in the UK, but was not part of the Australian research team who had designed the program.

### Topic guide

A semi-structured topic guide (see electronic supplementary materials) was devised by the research team (PW and KH) to facilitate discussion about participants’ perspectives of the program, what they liked and disliked, and how the program might be improved. Questions were open-ended, and probes used flexibly, encouraging participants to talk openly and provide an honest account. The questions were derived from an understanding of the online treatment literature, specifically the benefits and challenges associated with this method, in addition to anecdotal feedback from participants to therapists in the UK BRAVE-Online trial regarding their opinion of the program.

### Data analysis

Data were managed and stored using the computer software package NVivo, version 10 [38]. Analysis was undertaken in accordance with Braun and Clarke’s [39] description of reflexive thematic analysis which was chosen, because it is theoretically flexible and can be guided by a variety of concepts and is consistent with the ontogical and epistemological positioning of this research. Data were analysed following the six phases of reflexive thematic analysis [39]. To ensure familiarity with the data, audio recordings and transcripts were listened to and read multiple times. The researchers also referred back to the field notes as part of the analysis process but they were not coded and analysed as data. Transcripts were systematically analysed line-by-line, annotated and assigned codes. To ensure a credible account of the data, one researcher (KS) coded all the transcripts, and another (LS) coded one-third. Disagreements were resolved by discussion. KS organised the codes into clusters that reflected important patterns in the data. The themes were developed inductively from the data during coding. These preliminary themes and subthemes were then reviewed and revised during meetings with all authors to ensure they accurately represented the data.The product of this process was the classification of data into final specific and independent subordinate and superordinate themes.

To ensure that the analysis was conducted with rigour, we applied Guba and Lincoln’s four criteria [40, 41]. First, we ensured credibility by following Braun and Clarke’s [39] six-step process of analysis, having a clear process for coding, involving multiple raters, establishing a process for managing disagreements in coding, and incorporating an analysis of discrepancies within the data. Second, transferability was ensured by providing rich accounts of the detail of the data and considering the context of the data. Third, dependability was ensured by reporting the findings systematically and adopting an ‘auditing’ approach, i.e., keeping complete and detailed records of all stages of the research. Finally, confirmability was ensured by acknowledging the research team’s background, interests and biases and considering how this will have influenced the findings and research.

## Results

The analysis reported here focuses on the adolescents’ experiences of the treatment. Their perspectives come together to form two superordinate themes: (i) Usability of the program design; (ii) Putting techniques into practice. Endorsement of themes and sub themes can be found in Table 2.

### Theme 1: Usability of the program

#### An online program has benefits, but it is ‘not the same’ as face-to-face treatment

The convenience of completing sessions online ‘in your own time’ and not having to travel to the clinic that was ‘quite a way to go’ was seen positively by Jayne and Matthew, both of whom were very much improved at the end of treatment. However, connectivity and technical issues could get in the way of being able to access the program. ‘You could be in the middle of something quite important and the internet cuts out…it’s happened so many times’ (Zoe), or trying to run the program on an incompatible operating system meaning that ‘some of the things just wouldn’t come up so I couldn’t move on’ (George). Others, including those who were very much improved, were clear that they would have chosen to have sessions face to face with a therapist over online delivery, which was seen as ‘not the same’ (Isabelle).

*I just don’t really like online and stuff…like I used to do online lessons and I just I don’t feel like I was really taking it in as much as I would if you were actually, yeah, like real-life speaking to someone.* (Rosie)

*I think like the online stuff um was a lot less helpful than actually talking to people about things.* (Becky)

#### A lack of flexibility with format can interfere with engagement

The length of sessions and the large amount of reading involved was highlighted by the majority of participants. For some, the session length was fine, they liked ‘the way it was set out’ (Emma) and felt it had the ‘right amount of information’, particularly if they identified themselves as ‘quite a quick reader’ (Chloe) and were highly motivated.

*I was quite like keen to completely commit to it, I really wanted to do it so I think, I don’t think I minded putting a lot of time doing that.* (Becky)

For others, however, particularly those who did not consider themselves ‘a reading person’ (Zoe), the design of the sessions meant that they felt ‘held back’ (Isabelle), ‘weren’t taking it in as much’ (Rosie), or in some instances, ‘none of it went in’ (Zoe).

*It’s, we don’t necessarily like, teenagers, don’t really want to be reading a lot.* (Amelia)

The time taken to complete individual activities (e.g., games and problem-solving worksheets) and the overall session length meant that for some, it was experienced as ‘quite intense’ (Jayne; Isabelle), which meant that they ‘got a little bit tired’ (Emma), ‘started to lose interest a bit’ (Rosie) or couldn’t ‘be bothered’ (George; Isabelle) to carry on.

*Took about five years to do that bit!* (Zoe)

*I was like ‘oh my gosh, there’s like endless boxes. How many boxes are there?’* (Isabelle)

While repetition was built into the program to allow participants to practice and consolidate skills, for some, this was often perceived negatively.

*Because they kept doing it just it got kind of down, cus it just got boring.* (George)

*It could have I think been done in a more like concise way.* (Jayne)

The transdiagnostic design of the program meant that it could be helpful ‘understanding the different types of anxiety’ (Oliver), especially for those with comorbid anxiety disorders. However, more commonly, participants felt that learning about anxiety disorders that they did not experience wasn’t ‘relevant’ (Becky), that they ‘couldn’t relate’ (Jayne), that they did not want to ‘learn everything about everyone else’s one’ (Rosie) and that ‘it wasn’t that helpful’ (Emma), perhaps reflecting the low levels of co-morbidity within the sample.

#### The fun aspects are appreciated but the program feels ‘a bit young’

Participants reflected on the design of the program and the degree to which they felt it was appropriate for people of their age. It was clear that many of the participants appreciated the aspects of the program designed to be less formal, specifically the animations and the quizzes, which meant that doing the program was ‘not a chore’ (Clare).

*In a way it probably made me feel a bit better that it wasn’t like, like really serious (…), if it was presented in a like really formal way, I’d have been a bit like, intimidated almost.* (Jayne)

On the whole, it was the younger adolescents (e.g., Chloe; Emma; Amelia, all aged 13) who felt that the style and content of the program was appropriate for their age range, e.g., ‘it was really good cus it was like based around my age’ (Amelia). However, this was not restricted to younger adolescents—Lana (aged 17) also felt the program was appropriate for her age. Across the age range, the quizzes were seen as ‘useful’ and a fun way of consolidating the learning to ‘keep it in your brain’ (Lana).

Although one participant felt that the muscle relaxation was ‘a bit more for older people’ (Zoe), in all other instances where participants felt that the program was not at the right level, they felt the program was ‘quite tailor made to a younger audience’ (Isabelle). Aspects that were identified as being ‘a bit young’ (George; Rosie), ‘babyish’ (Clare), ‘childish’ (Oliver) and ‘patronising’ (Becky; Jayne) related to the visual images, games, sounds and music, as well as the use of rewards.

*I thought maybe they were again for a slightly younger audience. I think at one point there was a treasure chest.* (Becky)

Interestingly, some young people identified the program as being aimed at a younger age group ‘but at the same time…found it helpful’ (Isabelle).

*It got the message across and that was the most important thing.* (Jayne)

However, others found this harder to overlook.

*I think also the thing I was probably a bit over-sensitive to the whole kind of, it being for a younger audience thing because I felt a bit silly anyway.* (Becky)

#### Therapist’s support is important

Almost all the participants valued having a therapist ‘just sort of being there’ (Lana), ‘keeping track of things’ (Oliver) and feeling that there was ‘some sort of support’ (Jayne) to trouble-shoot any difficulties.

*They were always there to email you back like, they were really good if I did need something.* (Rosie)

*Having someone else as well that you can talk to about it. Yeah that was good.* (Emma)

Some adolescents did, however, highlight the difficulty of having feedback and any interaction with their therapist *after*, rather than *during*, the session. By the time they got to the end of the session, they would ‘be like I can’t really remember what I found difficult now’ (Rosie). Although participants were generally positive about the content and format of the individualised emails they received from their therapist, one person felt they were ‘a bit impersonal’ and involved ‘just more kind of reading’ (Clare) and others ‘didn’t look’ (Oliver) at them.

In general, participants felt it was ‘helpful to speak’ (George) to their therapist by telephone mid-way through treatment to help devise the exposure hierarchy, although some were ‘more scared’ (Oliver) doing this over the telephone than email. Having only received emails from their therapist up to this point meant that the phone call could be disconcerting.

*I found that went well but at the same time because I hadn’t met the person I was like ‘who am I talking to?’* (Isabelle)

Participants reflected on whether speaking via video-conferencing software could make this feel more personal. For some, this ‘would be better’ (Emma), while for others, this elicited anxiety about needing to ‘look okay’ (Chloe). Anxiety around therapist contact were not confined to those with social anxiety disorder. Having this session in person was also suggested as being better than a phone call, with ‘seeing your therapist more’ (Clare) seen as a way to improve the experience of treatment overall.

*Um that you could actually like see what they’re um saying and like if they needed to write stuff down then they can write it down for you like if you didn’t get it in a certain way.* (Amelia)

### Theme 2: Putting techniques into practice

#### The program provides an opportunity to gain understanding and see things differently

Psychoeducation was generally positively received by participants as the ‘scientific perspective’ (Jayne) allowed them to develop a ‘better understanding’ (Lana) of symptoms, challenge associated catastrophic beliefs and enable them to see that they were ‘normal’ (Chloe; Zoe; Lana), ‘not the only person that has it’ (Oliver) and feel less ‘alone’ (Rosie).

*It was just really good learning about like, I don’t know, feeling that you’re not going, I don’t know, not going crazy or going to die.* (Jayne)

This appeared to be particularly important when explaining physical symptomology.

*I kept getting shaky in classes or I’d just get randomly shaky and it’s normal, like I didn’t know before, I just thought I was really weird.* (Zoe)

*Fascinating…yeah cus then you know what starts it so you can try and calm yourself so it doesn’t get as bad and you can stay in the situation.* (Oliver)

Nevertheless, some young people (especially those who had had therapy before) felt that they ‘already knew’ (Emma) the information, suggesting it would be more beneficial for ‘someone who’s kind of new to it, like doesn’t really know what’s going on at all’ (Rosie).

Cognitive restructuring or ‘reality checking’ was seen as being a ‘useful’ (Matthew) strategy for dealing with anxiety, enabling participants to ‘put everything in perspective’ (Zoe) and see the situation from ‘another angle’ (Emma). Others reported that they tried to think positively in situations.

*Now like, if I’m worried about things I just think positive and then it just goes away.* (Chloe)

#### Doing exposure is important, but can be difficult

Learning about the role of avoidance in maintaining anxiety was seen as important, and participants talked about now being able to recognise when their instinct was to avoid a situation, and instead of avoiding, trying to expose themselves to their fears instead.

*I went to gym and I avoid doing this move because last time I did it I landed on my head and avoiding it made it worse…so I went back to the gym and tried it and it was fine.* (George)

*Once you’ve done it you don’t have to worry.* (Lana)

Most of the participants reflected on how devising an exposure hierarchy within the program and undertaking the steps as homework activities had been helpful (‘one of the best bits’—Amelia), enabling them to learn ‘it’s not actually that bad’ (George) and succeed in ‘conquering’ (Zoe) their fears.

*I just don’t like putting my hand up and things like that and that was one of my like steps and I’ve since that I’ve put my hand up a lot more…yeah, so it did help.* (Clare)

Breaking it down into steps through the exposure hierarchy was generally seen as helpful.

*It just helped me think of doing things in a better way, not panicking about it, kind of working it out, just doing one step then doing another step, it was easier doing it that way.* (Lana)

Nevertheless, as the exposure was undertaken in the absence of the therapist, it required participants to be ‘quite determined’ and this could be difficult if you were ‘having a bad day’ (Rosie). For some, there were difficulties identifying and setting up a meaningful hierarchy, especially for those with generalized anxiety disorder (Matthew; Chloe). Chloe ended up developing an exposure hierarchy with her therapist that was around her ‘staying at home on my own which I don’t like… and some of my friends don’t like it either’. She felt this was ‘just to test me’ and ‘not helpful at all’; this perspective appeared to be shared by her mum, who she reported ‘was like, ‘do I really have to go out’’. It could also be difficult to get the design of the hierarchy right in terms of not making the steps too easy or too difficult, although the therapist’s support could help with this.

*I think it was sort of planning a bit too far ahead (…) you don’t feel confident if you do really low things…it gradually helps when you get to harder things I think.* (Oliver)

*That was really helpful (…) cus like, I would have made bigger steps, so yeah she kind of divided them all up.* (Zoe).

#### Support for some components is mixed

Relaxation was perceived to have been beneficial by some participants (including all three boys) when they felt anxious.

*I often found that the deep breathing was the most useful.* (Matthew)

In particular, participants described using the relaxation CD or strategies to help with difficulties falling asleep (George; Chloe) or to manage stress, such as exams (Oliver). However, around half the participants reported that they ‘didn’t like it’ (Isabelle), were not ‘getting much from it’ (Emma) or ‘just didn’t use it’ (Jayne).

The use of rewards was also met with mixed responses. For older participants in particular (e.g., Becky, Emma; Chloe), achieving the steps on the exposure hierarchy was rewarding in itself (e.g., ‘I just felt proud after doing things’ – Oliver) and a reward ‘wasn’t necessary’ (Becky).

*I would like stand in a room with spiders because I wanted to get through my anxiety not because I wanted a reward.* (Chloe)

*When I went on [a ride at theme park] it was rewarding because I felt the thrill of doing it and now I love it.* (George)

This meant that some participants felt that that competing the activity to identify rewards was pointless, ‘just me sort of filling in boxes’ (Jayne). However, others enjoyed having a reward to aim for, as well as recognising the intrinsic reward of achieving the step (e.g., Oliver; Emma).

*I would have tried to do them anyway, but I still think it was a good thing to have.* (Emma)

For younger participants in particular, there was a feeling that a reward ‘gives you something to work towards’ (Matthew), acts as an incentive that ‘pushed me further’ (Amelia) and ‘encourages you to do it next time’ (Clare). Because completing the exposure steps was ‘so hard to do…you want to get something back for it’ (Rosie). For some, it even ‘got me through the week…gave me something to like look forward to’ (Zoe).

## Discussion

This study examined adolescents’ experiences of a therapist-supported online CBT programme for the treatment of anxiety disorders within CAMHS. Their perspectives formed two themes: (i) usability of the program, and (ii) putting techniques into practice. In relation to the usability of the program, although the convenience of online sessions was recognised as positive, many of the adolescents expressed a preference for face-to-face sessions with a therapist over online sessions with remote therapist support. The length of sessions and the large amount of reading involved was highlighted by most participants, and those who found reading more difficult could struggle with the format. The transdiagnostic nature of the content meant some elements of the programme were seen as less relevant and not beneficial. While many participants appreciated the ‘fun’ aspect of the program, others (particularly older participants), felt that certain aspects of the program (e.g., visual images, games, sounds and music, use of rewards) were not suitable for their age group. Participants valued having the support of a therapist, but there could be difficulties with the timing and nature of that support. In relation to putting the techniques into practice, psychoeducation and cognitive restructuring were generally seen positively, to better understand symptoms, especially intense bodily sensations, put things in perspective and think more ‘positively’. However, responses to the use of relaxation and rewards were much more mixed. Most of the participants reflected on how devising an exposure hierarchy within the program, breaking tasks into steps and undertaking the steps as homework activities had been helpful. Nevertheless, for some, there were difficulties identifying a focus for exposure and setting up effective and meaningful exposure hierarchies (Table 2).

**Table 2:** Endorsement of themes and subthemes

Themes	Matthew	George	Chloe	Isabelle	Zoe	Clare	Rosie	Lana	Emma	Oliver	Jayne	Becky	Amelia	Total
Theme 1: Usability of the program														
An online programme has benefits, but is not the same as face-to-face treatment	*	*	*	*	*		*				*	*	*	9
A lack of flexibility with format can interfere with engagement	*	*	*	*	*	*	*		*	*	*	*	*	12
The fun aspects are appreciated, but the program feels a bit young		*	*	*	*	*	*	*		*	*	*	*	11
Therapist’s support is important		*	*	*	*	*	*	*	*	*	*	*	*	12
Theme 2: Putting techniques into practice														
The program provides an opportunity to gain understanding and see things differently	*	*	*		*	*	*	*	*	*	*		*	11
Doing exposure is important, but can be difficult	*	*	*	*	*	*	*	*	*	*	*	*	*	13
Support for some components is mixed	*	*	*	*	*	*	*	*	*	*	*	*	*	13

The experiences of the adolescents in relation to the format of the treatment program was largely consistent with the broader literature. While there was some recognition of the convenience of the online format, a general preference for face-to-face sessions with a therapist rather than online sessions has been found in other studies involving adolescents from both CAMHS [42] and community populations [15]. In the current study, reasons appeared to relate to perceptions that face-to-face sessions would allow therapists to individualise content (for anxiety type, developmental level and individual differences, such as being ‘a reader’ or not), be more responsive to issues as they arose, and enable adolescents to take in the material better. This mirrors the results of a meta-synthesis of qualitative studies of online treatments for depression and anxiety across the age span [25], where they identified the need for programmes to be sensitive and responsive to the individual’s clinical needs, personal preferences and current functioning as one of the core themes. Studies with adults (e.g., [20]) have suggested that online programs may have high levels of acceptability, allowing ease of access and (often in contrast to attending face-to-face sessions) enabling individuals to access treatment without having to take time off work. However, it may be that for some adolescents, completing sessions in their own time (i.e., evenings/weekends) alongside homework, outside-school activities and leisure time is seen as burdensome and they may not consider missing school a problem; interestingly, Day, Carey and Surgenor [43] conducted a qualitative study with young people who had been seen in CAMHS and found that several reported being pleased to have avoided unpopular school lessons to attend appointments.

In general, the findings in relation to the most valued components of treatment was consistent with the current literature in terms of the relative effectiveness of different aspects of treatment. As well as psychoeducation and cognitive restructuring, most of the participants reported that designing an exposure hierarchy and undertaking the exposure steps had been beneficial. Recent quantitative and qualitative studies have demonstrated the importance of exposure in the treatment of anxiety disorders in young people [44–47]. Nevertheless, designing and undertaking meaningful exposure tasks was not always easy, especially for those with generalized anxiety disorder, where it may have been more difficult to identify situations avoided by the young person. These difficulties may not be confined to online treatment. Taylor et al. [47] also found that the young people in their study who had received face-to-face cognitive therapy for social anxiety disorder reported that exposure (through the form of behavioural experiments) had been the most salient and critical part of the treatment, but also widely reported as difficult for both the young person to complete and for the therapist to deliver. In the current study, participants were more mixed in their views of relaxation, with some reporting that they found it unhelpful. This is consistent with findings that the presence of relaxation within protocols is associated with less effective treatment [44, 46, 48]. For those who found relaxation helpful, this appeared to be at specific times, such as getting sleep or before an exam, suggesting that it is not necessary prior to and during exposure exercises, to be able to tolerate the treatment technique.

### Strengths and limitations

This is a robustly designed study that meets the criteria of the COREQ guidelines for rigorous reporting of qualitative research. Whilst there were differences in the experiences and perspectives of the adolescents, the themes described were consistent across all participants. We interviewed adolescents being treated within a routine NHS CAMHS setting, who were of a range of ages and primary diagnoses and had varying outcomes in treatment, which provided some diversity within the sample. Nevertheless, all participants took part in a randomised controlled trial and, therefore, are a subsample of those within routine services and as such, may not be typical CAMHS service users. Everyone in this sample had completed at least 80% of the treatment sessions and we were not able to interview any of the nine participants who had dropped out of treatment. It was notable that we were not able to recruit anyone who had a mood disorder prior to treatment (compared to nearly a quarter of the adolescents in the trial) and within this sample, only two of the adolescents had secondary anxiety disorders (compared to over half the adolescents who took part in the treatment trial). A comorbid mood disorder may have an impact on issues such as motivation and engagement with an online program. Perspectives on issues such as the transdiagnostic nature of the program (meaning that some content was irrelevant and not beneficial) may not have been shared by adolescents with comorbid anxiety disorders and, overall, the perspectives of the adolescents in the sample may be more positive than those who did not continue with the program. In addition, the participants who took part in both the trial and the subsequent qualitative interviews, were predominantly White British from relatively high socioeconomic backgrounds, and this is likely to limit generalization to other populations. Going forward, it will be crucial to better understand the experiences and needs of young people with greater psychiatric co-morbidity, greater ethnic diversity and those who have chosen to discontinue online treatments. All participants were interviewed face-to-face and this modality may also have had an impact on their views of the intervention, for example, their views around the benefits of face-to-face versus online sessions. All participants were interviewed after the 6-month follow-up assessment had taken place. Although all were shown the webpages as prompts to help with recall within the interview and some had completed more recent booster sessions following treatment, the gap from finishing treatment to conducting the interview may have influenced their ability to accurately recall their experiences. Finally, since this study was carried out, many clinical services have transferred to online modes of treatment delivery as a result of the COVID-19 pandemic. Similarly, adolescents are likely to have experienced lessons delivered remotely when they have not been able to attend school in person. These experiences may have changed how adolescents perceive online treatments and further research is necessary to understand their perspectives in a post-COVID world.

### Clinical and research implications

The findings have several clinical and research implications. They suggest that while some young people find the online format acceptable and effective, for others, especially where there are barriers to access (e.g., poor Wi-Fi, lack of devices, difficulties with concentration, reading or motivation difficulties), alternatives should be available. This is particularly important to consider given that as a result of the COVID-19 pandemic, many clinical services have moved to offering therapy remotely to individuals. As highlighted by the #iCBTLorentz Workshop Group [24], in the development of new online treatments, user engagement and the use of co-design is crucial from the outset to ensure that programs meet the needs of their users and have high levels of acceptability. There are huge challenges in designing a program that is acceptable and effective across anxiety disorders and with both younger and older adolescents and it may be that a ‘one size fits all’ approach simply cannot meet the needs of all individuals. Rather than following a linear program structure, there may be benefits to a structure that allows greater flexibility and responsiveness. This may involve modules which may be selected by either the young person or the therapist that suit their specific concerns or developmental needs. Modules could involve content that relates to particular anxiety disorders or comorbid difficulties, or treatment components that vary in their appeal depending on development/age or personal preference (e.g., rewards, games, etc.). Given the issues around reading, it will also be important to ensure that the overall program ensures that adolescents can access the material and remain engaged. Finally, it will be important to consider how best to involve the therapist in ways that meet the needs of young people and allow them to be responsive to problems as they arise.

## Conclusions

Online treatments provide an opportunity to increase access to evidence-based treatment for anxiety disorders in adolescents. This has been particularly evident over the past year as services have looked to ways to offer therapy remotely to individuals during the COVID-19 pandemic. Our findings highlight the potential utility for online treatments delivered with therapist support. However, they also highlight how issues with both the format and treatment components can impact the acceptability of a program. As such, further work is warranted to improve the acceptability of online treatments for adolescents with anxiety disorders.

## Supplementary Information

Below is the link to the electronic supplementary material.

Supplementary file1 (DOCX 28 KB)

## Data Availability

Research materials can be accessed by contacting the corresponding author.
