# Supplementary material for: The acceptability of a therapist-assisted internet-delivered cognitive behaviour therapy program for the treatment of anxiety disorders in adolescents: a qualitative study
Source: Eur Child Adolesc Psychiatry. 2021 Nov 8;32(4):661–73. doi: 10.1007/s00787-021-01903-6 (PMC8572655; doi:10.1007/s00787-021-01903-6)
Supplement: Supplementary file 1 — Supplementary file1 (DOCX 28 KB) [file 787_2021_1903_MOESM1_ESM.docx]

A qualitative study of adolescents’ experiences of a therapist-assisted Internet-delivered cognitive behaviour therapy program for the treatment of anxiety disorders

Katy Smart, Lydia Smith, Kate Harvey and Polly Waite

**Electronic Supplementary Materials**

**Appendix A: Topic guide**

1. What did you think about BRAVE overall?
2. Were there particular parts that stood out as important?
3. Were there things that you found useful?

Prompt: what was useful about it

1. Were there things that you found unhelpful?

Prompt: what was unhelpful about it

1. Were there any parts of BRAVE that you felt were too young or too old for you?

Prompt: What were they; did they get in the way of finding sessions helpful – if so, how

1. I’d now like to show you the sessions that you did and when we look through them, please can you tell me which bits you liked (or thought were helpful) and which bits you didn’t like (or didn’t think were helpful)? (show webpages to adolescent)

Prompt: what was helpful/unhelpful about that?

1. Are there any things that you would suggest that you think would improve the sessions?
2. Do you have any thoughts about how we could improve the BRAVE ladder?
3. What did you think about having the support of a therapist alongside the written materials?
4. What did you think about the emails you got from the therapist?
5. What did you think about talking to the therapist on the phone to plan your BRAVE ladder?
6. How did you find the level of support the therapist gave you?
7. When the therapist reminded you to do sessions, did they give you the right level of support? If not, how could they improve this?
8. This was the structure of the sessions (show sheet to adolescent). Would you change this in any way?
9. There were 10 sessions in total – would you change this?

Prompt: In what way

1. There were 2 booster sessions – did you do them?

Prompt: If not, why not; if yes, were these helpful and in what way

1. Finally, is there anything we haven’t covered that you would like to add about your experience of doing BRAVE?

|  |
| --- |

**Appendix B: Consolidated criteria for reporting qualitative studies (COREQ): 32-item checklist**

| **No. Item** | **Guide questions/description** | **Reported on Page #** |
| --- | --- | --- |
| **Domain 1: Research team and reﬂexivity** |  |  |
| *Personal Characteristics* |  |  |
| 1. Interviewer/facilitator | Which author/s conducted the interview or focus group? | Page 12 |
| 2. Credentials | What were the researcher’s credentials? E.g. PhD, MD | Page 12 |
| 3. Occupation | What was their occupation at the time of the study? | Page 12 |
| 4. Gender | Was the researcher male or female? | Page 12 |
| 5. Experience and training | What experience or training did the researcher have? | Page 12 |
| *Relationship with participants* |  |  |
| 6. Relationship established | Was a relationship established prior to study commencement? | Page 12 |
| 7. Participant knowledge of the interviewer | What did the participants know about the researcher? e.g. personal goals, reasons for doing the research | Page 11 |
| 8. Interviewer characteristics | What characteristics were reported about the interviewer/facilitator? e.g. Bias, assumptions, reasons and interests in the research topic | Page 12 |

| **Domain 2: study design** |  |  |
| --- | --- | --- |
| *Theoretical framework* |  |  |
| 9. Methodological orientation and Theory | What methodological orientation was stated to underpin the study? e.g. grounded theory, discourse analysis, ethnography, phenomenology, content analysis | Page 13 |
| *Participant selection* |  |  |
| 10. Sampling | How were participants selected? e.g. purposive, convenience, consecutive, snowball | Page 10 |
| 11. Method of approach | How were participants approached? e.g. face-to-face, telephone, mail, email | Page 10 |
| 12. Sample size | How many participants were in the study? | Page 9 |
| 13. Non-participation | How many people refused to participate or dropped out? Reasons? | Page 10 |
| *Setting* |  |  |
| 14. Setting of data collection | Where was the data collected? e.g. home, clinic, workplace | Page 12  . |
| 15. Presence of non-participants | Was anyone else present besides the participants and researchers? | Page 12 |
| 16. Description of sample | What are the important characteristics of the sample? e.g. demographic data, date | Page 9 & 27-28 |
| *Data collection* |  |  |
| 17. Interview guide | Were questions, prompts, guides provided by the authors? Was it pilot tested? | No |
| 18. Repeat interviews | Were repeat interviews carried out? If yes, how many? | No |
| 19. Audio/visual recording | Did the research use audio or visual recording to collect the data? | Page 9 |
| 20. Field notes | Were ﬁeld notes made during and/or after the interview or focus group? | Page 12 |
| 21. Duration | What was the duration of the interviews or focus group? | Page 12 |
| 22. Data saturation | Was data saturation discussed? | Page 10 |
| 23. Transcripts returned | Were transcripts returned to participants for comment and/or correction? | No |
| **Domain 3: analysis and ﬁndings** |  |  |
| *Data analysis* |  |  |
| 24. Number of data coders | How many data coders coded the data? | Page 13 |
| 25. Description of the coding tree | Did authors provide a description of the coding tree? | No |
| 26. Derivation of themes | Were themes identiﬁed in advance or derived from the data? | Page 13 |
| 27. Software | What software, if applicable, was used to manage the data? | Page 13 |
| 28. Participant checking | Did participants provide feedback on the ﬁndings? | No |
| *Reporting* |  |  |
| 29. Quotations presented | Were participant quotations presented to illustrate the themes/ﬁndings? Was each quotation identiﬁed? e.g. participant number | Page 14-22 |
| 30. Data and ﬁndings consistent | Was there consistency between the data presented and the ﬁndings? | Page 14-22 |
| 31. Clarity of major themes | Were major themes clearly presented in the ﬁndings? | Page 14-22 |
| 32. Clarity of minor themes | Is there a description of diverse cases or discussion of minor themes? | Page 14-22 |
